# Intratumoral Androgens and Genetic Variants Driving Therapy Resistance in Prostate Cancer

**DOI:** 10.34133/research.1128

**Published:** 2026-02-24

**Authors:** Junjiang Ye, Yandong Xie, Jie Wang, Ruicheng Wu, Dengxiong Li, Koo Han Yoo, Dilinaer Wusiman, William C. Cho, Zhaojie Lyu, Dechao Feng

**Affiliations:** ^1^ Urology & Nephrology Center, Department of Urology, Zhejiang Provincial People’s Hospital (Affiliated People’s Hospital), Hangzhou Medical College, Hangzhou, 310014 Zhejiang, China.; ^2^ Department of Urology, Institute of Urology, West China Hospital, Sichuan University, Chengdu, Sichuan, China.; ^3^ Department of Urology, Nanfang Hospital, Southern Medical University, Guangzhou, Guangdong, China.; ^4^ Division of Surgery and Interventional Science, University College London, London W1W 7TS, UK.; ^5^ Department of Urology, The First Affiliated Hospital of Zhejiang Chinese Medical University (Zhejiang Provincial Hospital of Chinese Medicine), Hangzhou, Zhejiang Province, China.; ^6^ Department of Urology, Kyung Hee University, Seoul, South Korea.; ^7^ Purdue Institute for Cancer Research, Purdue University, West Lafayette, IN 47907, USA.; ^8^ Department of Clinical Oncology, Queen Elizabeth Hospital, Hongkong SAR, China.; ^9^ Department of Urology, Institute of Precision Medicine, Peking University Shenzhen Hospital, Shenzhen 518036, China.

## Abstract

The persistence of castration-resistant prostate cancer (CRPC) despite androgen deprivation therapy and androgen receptor (AR) signaling inhibition underscores the need to elucidate resistance mechanisms. The AR signaling pathway plays a central role in the development of prostate cancer. Metabolic reprogramming of androgen synthesis and aberrant activation of AR signaling collectively drive CRPC development. Under therapeutic pressure, AR signaling adapts through AR amplification, ligand-binding domain mutations, splice variants, and alternative activation by cytokines/growth factors, maintaining AR transcriptional activity in low-androgen environments. Concurrently, somatic alterations (like PTEN loss) and crosstalk with key pathways such as PI3K/AKT, coupled with the evolving multifocal spatial heterogeneity, further complicate the role of AR signaling in CRPC treatment resistance. Innovations in single-cell and spatial technologies reveal tumor heterogeneity and lineage plasticity governed by genetic and epigenetic alterations. Current therapeutic innovations, including approaches such as CYP11A1 inhibition, targeting of the AR N-terminal domain, and bipolar androgen therapy, are showing promise in clinical trials. Overcoming CRPC effectively requires cotargeting androgen/AR-associated pathways and suppressing lineage plasticity through dynamic monitoring and precision interventions.

## Introduction

With population aging intensifying, the age-standardized disability-adjusted life year rate for prostate cancer (PCa) was 217.83 per 100,000 population in 2021, and new cases are projected to reach 2.58 million worldwide by 2046 [1]. While localized PCa can be effectively managed through active surveillance or radical treatments (surgery or radiotherapy), the prognosis deteriorates substantially upon progression to advanced stages [2,3]. Androgen deprivation therapy (ADT), combined with androgen receptor (AR) signaling inhibitors (ARSIs), constitutes the standard systemic treatment for advanced PCa, suppressing tumor growth by reducing circulating testosterone to castration levels and attenuating intracellular AR signaling, and demonstrating significantly delayed disease progression [4]. However, therapeutic resistance is inevitable, with the majority of patients eventually developing castration-resistant prostate cancer (CRPC), a highly aggressive subtype characterized by poor prognosis and limited treatment efficacy [5].

Mechanistically, AR signaling pathway plays a profound role in PCa initiation, progression, and therapeutic resistance [6]. The vast majority of primary PCa tumors express AR and exhibit marked dependence on AR signaling for cellular proliferation and survival [7]. The mechanisms underlying CRPC resistance are multifaceted, with sustained intratumoral androgen levels and persistent AR pathway activation identified as pivotal factors [5]. Under endocrine therapy pressure, heterogeneous alterations in the AR gene—including amplification, mutations, and splice variant generation—can perpetuate AR pathway activation [8]. Additionally, the enrichment of AR-negative or AR-low (AR^−^/lo) cell populations contributes significantly to therapeutic resistance [8,9]. Enhanced functionality of genes directly involved in androgen synthesis (like AKR1C3) further elevates intratumoral androgen levels, sustaining AR signaling activity [10]. Concurrently, genetic aberrations in key tumor suppressors (like *PTEN* and *RB1*) and dysregulation of signaling pathways [like phosphatidylinositol 3-kinase (PI3K)] interact with AR signaling to form a complex resistance network, directly or indirectly influencing AR pathway stability and cellular genealogies [11,12]. The evolving tumor heterogeneity of these alterations, driven by multifocal PCa stem cells (PCSCs), further exacerbates the complexity of drug resistance [13].

Thus, comprehensive elucidation of the AR-centric resistance mechanisms—spanning intratumoral androgen metabolism, AR genetic alterations, multidimensional pathway crosstalk, and spatiotemporal heterogeneity—is imperative for developing precision therapies for CRPC. This review systematically synthesizes current knowledge on these interconnected drivers of therapeutic resistance, thereby providing a theoretical foundation for future individualized treatment approaches against CRPC (the core references cited in this review are provided in Table S1).

## The Regulation of Intratumoral Androgen Levels and Metabolic Mechanisms

More than 90% of testosterone is secreted by the testes, with the remainder primarily derived from adrenal secretion [14]. Under ADT for PCa, detectable levels of active androgens persist within tumor tissues [15]. This phenomenon highlights the critical roles of (a) local de novo synthesis in the prostate, (b) uptake and conversion of adrenal-derived androgen precursors, and (c) intratumoral regulation of androgen metabolism, in maintaining active androgen levels within tumors under low circulating androgen conditions [15,16].

### Mechanisms of androgen synthesis

**Fig. 1.**
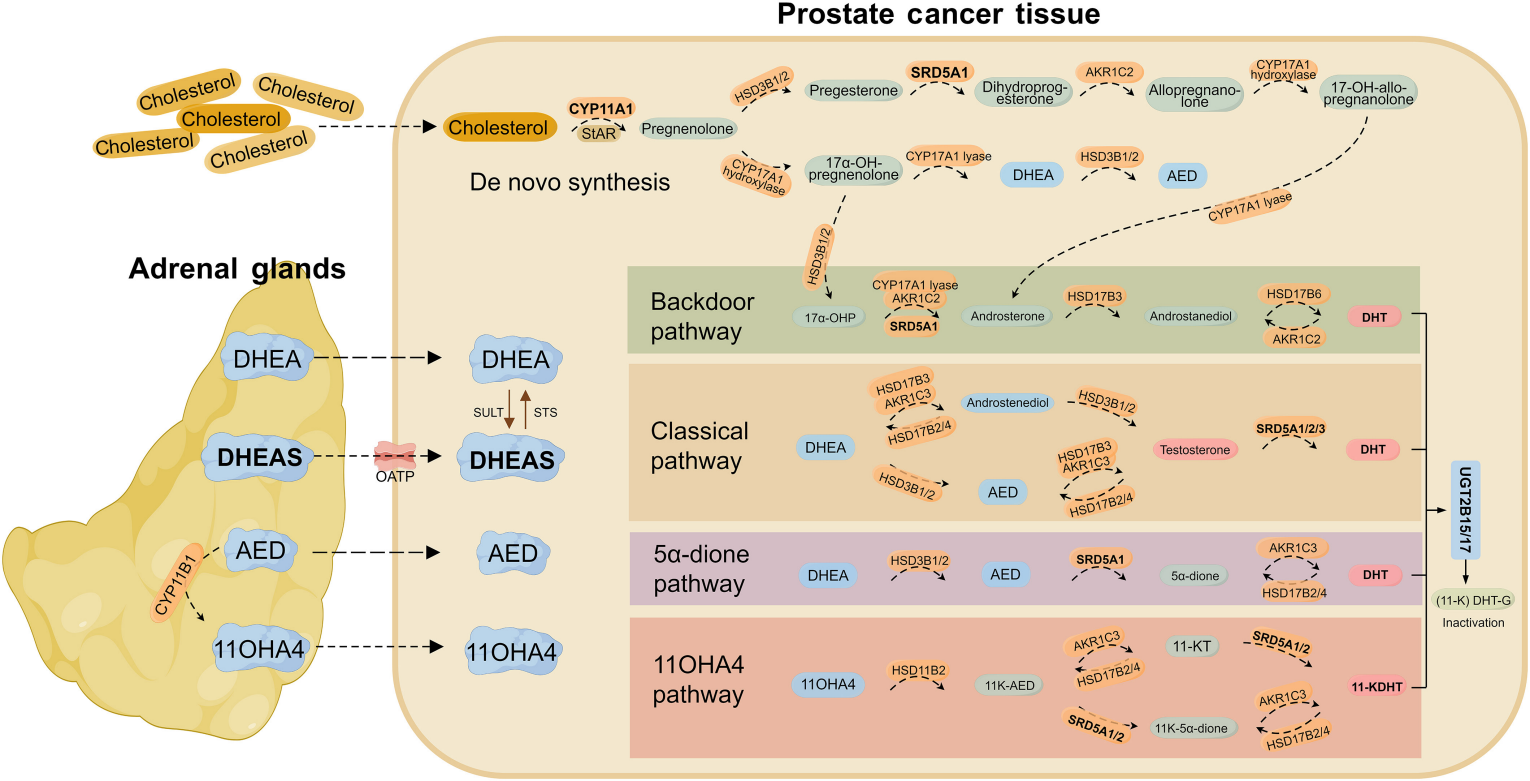
Intratumoral androgen synthesis pathways in CRPC from de novo and adrenal precursors. De novo synthesis: In CRPC tumors, steroidogenic enzymes enable androgen production from cholesterol. The steroidogenic acute regulatory protein (StAR) initiates de novo androgen synthesis by mediating cholesterol transport into mitochondria. Expression of the steroidogenesis enzymes or protein can be up-regulated via paracrine signaling (e.g., by IL-6 and IGF-2) [51,52]. Adrenal precursor-based synthesis: PCa cells utilize adrenal androgen precursors—DHEAS, dehydroepiandrosterone (DHEA), androstenedione (AED), androstenediol, and 11OHA4, to synthesis androgens. DHEA-S requires active transport via organic anion-transporting polypeptides, while others diffuse passively [23]. Within PCa tissues, these precursors are converted to active androgens through multiple pathways. Classical pathway: Androgen precursor DHEA is first converted to AED/androstenediol, which is subsequently metabolized to testosterone and DHT [17,23]. Backdoor pathway: 17α-OHP is first catalyzed by SRD5A1 and then undergoes sequential enzymatic reactions to directly yield DHT, bypassing testosterone formation. 5α-dione pathway: AED is initially converted to 5α-dione by SRD5A1 and then transformed into DHT. 11OHA4 pathway: 11OHA4 can be converted to 11-ketotestosterone (11-KT) or 11-ketodihydrotestosterone (11-KDHT) through enzymes including HSD11B2 and AKR1C3 [23]. Finally, active androgens can be inactivated through glucuronidation mediated by uridine diphosphate-glucuronosyltransferase 2B15/17 (UGT2B15/17) [47,48]. The figure was created by Figdraw.

The persistence of intratumoral androgens in CRPC is largely sustained through 4 major metabolic pathways: the classical, backdoor, 5α-androstanedione (5α-dione), and hydroxyandrostenedione (11OHA4) pathways (Fig. 1).

Endogenous de novo androgen synthesis utilizes cholesterol as precursor. In the classical pathway, cholesterol is converted to pregnenolone by CYP11A1, followed by sequential catalysis involving CYP17A1, HSD3B1/2, AKR1C3, and SRD5A to produce testosterone and ultimately dihydrotestosterone (DHT) [17,18]. Although some studies identified the lack of StAR, CYP11A1, and CYP17A1 in PCa tumor tissues, the up-regulation of downstream steroidogenic enzymes like SRD5A1 and AKR1C3 enhances utilization of CYP17A1 downstream androgen precursors [19–21]. Such adaptation allows tumors to efficiently convert adrenal-derived precursors into potent androgens under castrate conditions. Notably, SRD5A1 exhibits a substrate preference for 17-hydroxyprogesterone (17α-OHP) over androstenedione (AED), and prefers AED over testosterone [21,22], which facilitates the backdoor pathway—a bypass route that directly generates DHT without testosterone as an intermediate [23,24]. Furthermore, in CRPC, due to the potential deficiency of HSD17B3 and the low catalytic efficiency of AKR1C3 toward AED [21,25], dehydroepiandrosterone (DHEA) preferentially converts to AED, which is then metabolized to DHT via 5α-androstanedione (5α-dione), forming the 5α-dione pathway [26]. Notably, DHEA can be metabolized by HSD17B into 5-androsten-3β,17β-diol, an isomer of androstenediol and a potent agonist of estrogen receptor β, which may mediate tumor-suppressive effects [27].

Following ADT, circulating testicular-derived androgens are markedly reduced, and adrenal-secreted androgen precursors—including dehydroepiandrosterone sulfate (DHEAS), DHEA, AED, androstenediol, and 11β-hydroxyandrostenedione (11OHA4)—become the primary exogenous sources of intratumoral androgens [23,28] (Fig. 1). PCa cells up-regulate organic anion-transporting polypeptides to uptake DHEAS, the major adrenal-derived precursor, which is subsequently hydrolyzed by steroid sulfatase to generate DHEA, thereby entering the androgen synthesis pathway [29]. These precursors can be metabolized through the aforementioned pathways, with the 5α-dione pathway may dominate due to its metabolic efficiency and simplicity [21,25,26,30]. Additionally, the 11OHA4 pathway is activated in CRPC, yielding 11-oxygenated androgens such as 11-ketotestosterone and 11-ketodihydrotestosterone [25,31]. These androgens exhibit AR-activating potency comparable to canonical androgens, are resistant to inactivation, and reach physiologically relevant concentrations in CRPC tissues [32].

Although CYP17A1-targeted agents like abiraterone effectively suppress androgen precursor conversion, tumor cells frequently develop resistance by compensatory up-regulation of the nonclassical pathways, coupled with mechanisms such as progesterone accumulation, leading to metabolic reprogramming of androgen axis in CRPC [20,25,33,34]. These findings highlight the necessity of tailoring therapeutic strategies for CRPC by precisely inhibiting key enzymes involved in intratumoral androgen synthesis and metabolism to block androgen regeneration.

### Regulatory mechanisms of androgen metabolism

The regulation of androgen metabolism is pivotal in sustaining intracellular androgen levels and AR signaling, thereby driving resistance to endocrine therapies in CRPC. This regulation operates through multiple interconnected mechanisms, including cholesterol and lipid metabolism, epigenetic modifications, and influences from the tumor microenvironment (TME), all of which adapt under therapeutic pressure to maintain androgen availability.

Aberrant cholesterol accumulation provides abundant substrates for androgen synthesis, with levels significantly elevated in CRPC (with nuclear concentrations reaching twice the normal level) [35,36]. Sterol regulatory element-binding proteins 1/2 (SREBP1/2) are crucial transcription factors governing cholesterol metabolism, regulating the transcription of key cholesterol synthesis genes such as 3-hydroxy-3-methyl-glutaryl-CoA reductase (*HMGCR*) and fatty acid synthase [37]. Under androgen deprivation, SREBP1 dissociates from AR and translocates to the nucleus, promoting steroidogenic gene expression [38]. AR and PI3K/AKT/mammalian target of rapamycin (mTOR) signalings further up-regulate SREBP2 to amplify cholesterol production and further androgen synthesis [39]. Statins, which inhibit HMGCR to reduce cholesterol synthesis, have been shown to suppress androgen metabolism and overcome drug resistance in preclinical models. *SPOP* missense is frequently accompanied by *CHD1* deletion [40]. In PCa cells harboring *SPOP* mutations (about 12% prevalence), CHD1 deficiency leads to increased SREBP2 expression, promoting intratumoral cholesterol and androgen synthesis [40,41]. As *SPOP* mutations are associated with AR protein accumulation, *CHD1* deletion results in robust AR signaling activation [42]. Atorvastatin was demonstrated to block this compensatory pathway, revealing potential antitumor effects with abiraterone [40]. A meta-analysis further supports the potential clinical value of statins, showing they are related to improved survival in patients with advanced PCa [43].

Epigenetic mechanisms dynamically regulate androgen metabolism to maintain intratumoral androgen levels. Under high androgen conditions, the demethylase LSD1 suppresses AR expression by mediating demethylation of H3K4me1/2, and involved in suppressing androgen-synthesizing enzymes ( AKR1C3 and HSD17B6) [44]. Conversely, in low-androgen environments, phosphorylated SREBP1 promotes histone H2AK130ac enrichment to enhance its own transcription and up-regulate cholesterol synthesis genes, such as *HMGCS1* [38]. Exogenous melatonin supplementation shows potential against CRPC in preclinical studies [45]. Melatonin has been reported to promote DNA methyltransferase 1 deacetylation, relieving repression of carboxylesterase 1, which reduces lipid accumulation and indirectly down-regulates steroidogenic enzymes (CYP11A1 and STARD4) [46]. Further in vitro tests demonstrated that melatonin reversed enzalutamide resistance by reducing lipid accumulation and inhibiting intratumoral androgen synthesis [46]. Additionally, the glucuronidation enzymes UGT2B15 and UGT2B17 irreversibly inactivate androgens, yet their expression is suppressed by androgens themselves, epidermal growth factor (EGF), and miR-376c [47–49]. This regulation of UGT2B15/17 creates a self-reinforcing cycle of androgen accumulation.

The PCa microenvironment further regulates local androgen synthesis through stromal–epithelial paracrine interactions and signaling pathways of diverse secretory factors. PCa-derived stromal cells could exhibit high expression of SRD5A [50] and secrete factors such as interleukin-6 (IL-6) and insulin-like growth factor-2 (IGF-2), which up-regulate enzymes like StAR, CYP11A1, CYP17A1, and AKR1C3 in tumor cells, enhancing de novo androgen synthesis [51,52]. IL-6 treatment has been shown to increase testosterone synthesis in LNCaP cells by 2-fold [52]. Additionally, insulin, luteinizing hormone, and paracrine factors like fibroblast growth factor-2 and hepatocyte growth factor were also implicated in the potential regulation of intratumoral androgen metabolism in PCa tissues [53–55]. Cancer-associated fibroblasts (CAFs), as the most abundant stromal cells, closely interact with PCa through paracrine metabolites, regulating androgen accumulation. PCa cells recruit CAFs by secreting factors such as testosterone and nerve growth factor, while CAFs promote tumor progression through androgen-dependent extracellular matrix remodeling mediated by the AR/filamin A complex [56]. Recent studies have revealed that CAFs could secrete glucosamine and ADAM9, which up-regulate HSD3B1 and AKR1C3 expression in PCa cells, respectively [57,58]. Furthermore, microenvironmental chronic hypoxia in prostate tumors induces hypoxia-inducible factor-2α accumulation to transactivate *HSD3B1*, further fueling androgen synthesis [59]. These regulatory mechanisms ultimately promote androgen synthesis, driving the sustained progression of CRPC.

## AR Signaling Pathway and AR Variants

The maintenance of sufficient intratumoral androgen levels (as discussed in the previous section) provides the ligand foundation for the sustained activation of the AR. However, as the disease progresses and therapeutic pressure increases, alterations in the AR pathway itself constitute another central pillar driving drug resistance.

### Canonical AR signaling pathway

**Fig. 2.**
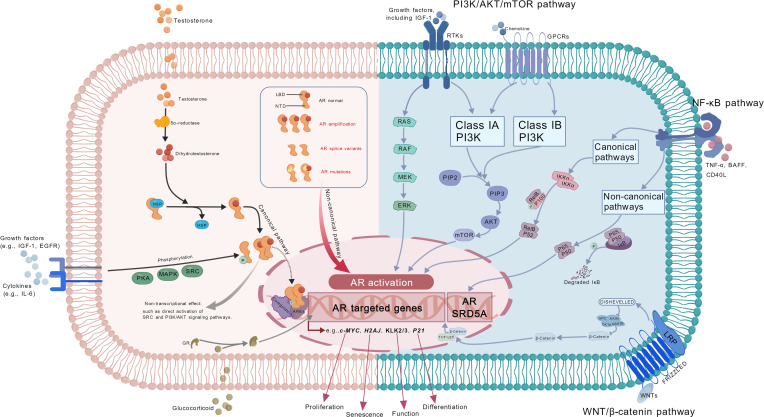
Androgen signaling pathway-centric crosstalk network in CRPC. Androgen binding to AR induces dissociation of heat shock proteins (HSPs) and AR nuclear translocation, regulating functionally diverse genes. AR alterations, including AR amplification, point mutations, and splice variants, enable sustained AR signaling under endocrine therapy pressure. Growth factors and cytokines could activate AR transcription via phosphorylation pathways including PKA, MAPK, and SRC. Additionally, AR can rapidly promote cell proliferation through nongenomic activation of SRC and PI3K/AKT signaling pathways [106]. Cross-pathway interactions: The PI3K/AKT pathway is activated through class IA PI3Ks (p110α/β/δ) and class IB PI3Ks (p110γ) with the involvement of receptor tyrosine kinases (RTKs) and G protein-coupled receptors (GPCRs), promoting AR signaling [106]. The NF-κB pathway modulates AR signaling through both canonical and noncanonical pathways: canonically, via p50/p65 dimers that enhance *AR* expression; noncanonically, through p52 binding to the AR N-terminal domain to potentiate transcriptional activity [112]. Additionally, AR can increase NF-κB pathway activity by promoting IκBα degradation [115]. The WNT/β-catenin pathway regulates AR signaling via β-catenin, which functions as a transcriptional coactivator [106]. The figure was created with BioGDP [163].

The canonical AR pathway plays a pivotal role in the development of PCa [8] (Fig. 2). AR is an intracellular steroid hormone-activated transcription factor. In its inactive state, AR predominantly binds to chaperones such as heat shock proteins [60]. Upon binding of androgens to the ligand-binding domain (LBD) of AR, a conformational change is induced, leading to nuclear translocation of AR and its subsequent binding to androgen response elements. This process regulates genes associated with cell proliferation (including *c-MYC*), senescence (including *H2AJ*), and prostate function (including *KLK2/3*) [61,62].

In certain cellular contexts, such as PC3 cells, AR may also exhibit tumor-suppressive effects by inhibiting proliferation via transactivating *P21*, and Retinoblastoma-1 (RB1) can assist in the tumor-suppressive function of AR [8]. Interestingly, in clinical practice, supraphysiological androgen therapy, such as bipolar androgen therapy (BAT), has emerged as a novel therapeutic strategy for advanced PCa in clinical trials. In the RESTORE study, enzalutamide-resistant patients were subjected to BAT with high-dose testosterone cypionate and then were rechallenged with enzalutamide following progression on BAT. The BAT achieved a PSA_50_ (50% decrease in prostate-specific antigen level) response rate of 30% [63]. Notably, in the enzalutamide–BAT–enzalutamide sequence, 68% regained PSA response to enzalutamide [63]. The recent phase II ExBAT trial (NCT04558866) further confirmed the substantial clinical efficacy of BAT [64].

### Activation of noncanonical AR signaling pathways

In treatment-naïve PCa, the vast majority of tumor cells are AR^+^ and rely on the canonical AR signaling pathway for growth. While androgens levels decline sharply under ADT pressure, PCa cells adapt and sustain AR signaling by activating noncanonical pathways, and AR bypass signaling, which enable persistent AR activation in low- or no-androgen environments and drive CRPC development (Fig. 2).

Hypoandrogenic conditions alleviates negative feedback inhibition on AR expression [44]. Tumor cells enhance AR expression through *AR* gene and upstream enhancer region copy number gain, and mRNA up-regulation, thereby amplifying signal output from residual low-concentration ligands [65–67]. *AR* amplification (observed in 50% to 85% of CRPC) heightens PCa sensitivity to androgens, representing both an early resistance hallmark and a core adaptive mechanism for CRPC survival in low-androgen milieus [65,68,69]. Furthermore, high AR expression enables AR antagonists (like bicalutamide) to recruit activating coregulators (like steroid receptor coactivator-1) while reducing nuclear receptor corepressor binding, thereby paradoxically activating part of androgen-sensitive genes (such as *PSA* and *KLK2*) and conferring partial agonist properties to antagonists [70,71].

AR splice variants (AR-Vs), particularly AR-V7 (constituting over 80% of CRPC cases in some studies), frequently lack LBD integrity and play pivotal roles in androgen-independent signaling and drug resistance [68,69,72]. Aberrant AR splicing is driven by multiple factors, including full-length AR (AR-FL) up-regulation, dysfunction of splicing factors, and epigenetic reprogramming [9,73,74]. Latrophilins (LPHNs) serve as critical downstream effectors of AR-V7 signaling [75]. LPHN activation induces Janus kinase 2 (JAK2)/signal transducer and activator of transcription (STAT3) phosphorylation and BCL-2 overexpression, thereby bypassing canonical AR signaling to drive tumor progression [75]. Overcoming AR-Vs resistance effectively requires targeting of AR-FL, AR-Vs, and AR-Vs downstream effectors like LPHNs. Multiple drugs, including therapeutic vaccines targeting AR splice variants, are currently under development [76–79].

*AR* mutations frequently occur in the LBDs (e.g., T877A, H874Y, and F877L), with a about 15% to 20% incidence in CRPC patients [9,80]. Mutant LBDs alter AR conformation and affinity, rendering antagonists ineffective and enabling AR activation even by non-androgenic ligands, including antagonists [80]. This underlies AR antagonist withdrawal syndrome [81,82]. For instance, under enzalutamide pressure, clonal expansion of AR-F877L mutants could induce an agonist-like active conformation, attenuating antagonist response and potentially converting enzalutamide into a partial agonist [83]. Enhancing the efficacy of AR antagonists against AR point mutations is a key research focus, with novel AR antagonists such as chrysin currently under investigation [84].

Additionally, growth factors (including IGF-1 and EGF) and cytokines (including IL-6) can activate AR transcription by promoting its phosphorylation via downstream kinases like protein kinase A (PKA), mitogen-activated protein kinase (MAPK), and SRC [85–87] (Fig. 2). Progesterone can even directly activate AR in a ligand-binding way [33]. Additionally, AR and glucocorticoid receptor (GR) share nearly 50% of direct target genes at the transcriptome level [88]. Therefore, GR agonists (like dexamethasone) can activate AR-sensitive target genes (such as *KLK2* and *FKBP5*) independently of AR pathway [88]. Although AR suppresses GR transcription by binding to GR introns, antiandrogen therapy induces about more than 2-fold GR expression up-regulation [88]. AR alternative signaling represents a critical mechanism for PCa progression under AR inhibition, illuminating CRPC development and therapeutic resistance.

## Multidimensional Drug Resistance Network of AR

PCa exhibits extensive genetic alterations that interact with core signaling pathways, forming an AR-centric multidimensional drug resistance network (Fig. 2). These alterations drive tumor progression and drug resistance by modulating AR signaling, activating AR compensatory survival pathways, or inducing epigenetic reprogramming to drive lineage plasticity, collectively escalating therapeutic complexity (Table 1).

**Table 1.** Current progress in promising clinical trials of novel AR pathway-related and AR-independent PCa-targeting drugs.

Therapeutic strategies	Pathway mechanisms	Therapeutic interventions	Treatment mechanisms	Clinical trials, ID (treatment plans)	Therapeutic efficacy
AR-targeting strategies	CYP11A1 inhibition blocks initial steroid synthesis [164]	ODM-208	Targeted inhibition of CYP11A	Phase I/II CYPIDES, NCT03436485 (ODM-208 monotherapy)	In mCRPC patients with a median follow-up of 5.8 months, the PSA_50_ rate was 53.3% in patients with AR mutation in phase 2 [164]
	High androgens induce AR hyperactivation, causing DNA damage and cell cycle arrest, and suppress AR and its variants [63]	BAT	Cell cycle interference, DNA damage, down-regulation of AR alterations, restoration of AR pathway therapeutic sensitivity [63]	Phase II ExBAT, NCT04558866 (alternating sequential administration of BAT and darolutamide)	Among mCRPC patients post-abiraterone treatment, the median rPFS was 9.0 months, with a PSA_50_ rate of 16.7% and median OS of 23 months [64]
	AR variants (amplification, LBD mutations, splice variants) sustain AR signaling in low-androgen environments [80]	BMS-986365	PROTAC BMS-986365 binds to the AR’s LBD, inducing AR degradation via ubiquitin–proteasome pathway, and locking AR in inactive conformation [165]	Phase I, NCT04428788 (BMS-986365 monotherapy)	In mCRPC patients with a median follow-up of 17.7 months, the overall PSA_50_ rate was 32%, with rPFS of 6.3 months; the AR LBD mutation subgroup exhibited a PSA_50_ rate of 55%, with rPFS of 6.3 months [165]
		ARV-766	PROTAC ARV-766 targets the LBD of AR (including the LBD mutations L702H, H875Y, and T878A) for AR degradation [166]	Phase I/II, NCT05067140 (ARV-766 monotherapy)	For mCRPC patients with AR LBD mutations, the PSA_50_ rate reached 50.0% [166]
	AR NTD mediates DNA binding and transcription; tumors with NTD but no LBD resist antiandrogen therapies [6]	EPI-7386	Third-generation ARSI EPI-7386, targets AR NTD to block transcription, independent of LBD status [167]	Phase I/II, NCT05075577 (EPI-7386 plus enzalutamide versus enzalutamide)	In mCRPC patients, the PSA_90_ rate of 13/16 patients was 81%. 5/16 evaluable patients showed measurable disease, with 3/5 (60%) partial response and 2/5 (40%) stable disease [167]
AR-related strategies	PI3K/AKT pathway promotes progression via AKT-mediated apoptosis suppression, mTOR-involved metabolic reprogramming, and reciprocal crosstalk with AR signaling [107]	Ipatasertib	Selective inhibition of AKT1/2/3	Phase III IPATential150, NCT03072238 (ipatasertib plus abiraterone versus placebo plus abiraterone)	In mCRPC patients with a median follow-up of 33.9 months, patients with NGS-detected PTEN deletions were associated with prolonged OS (36.8 months, HR = 0.76), as were PIK3CA/AKT1/PTEN alterations (37.1 months, HR = 0.70) [168]
	PARP-1/2 participates in single-strand break repair; BRCA1/2 participates in double-strand break repair; defects increase genomic instability [99,103]	Olaparib	Inhibition of PARPs in BRCA mutation patients, inducing lethal DNA double-strand breaks	Phase III PROpel, NCT03732820 (olaparib plus abiraterone versus placebo plus abiraterone)	mCRPC patients in the experimental arm achieved rPFS of 25 months (HR = 0.66), significantly superior to controls. The BRCA-mutated subgroup demonstrated further improved rPFS (HR = 0.24) and OS (HR = 0.30) [169]
AR-independent strategies	EZH2 catalyzes H3K27me3 to repress tumor suppressors, and promotes *AR* transcription directly, driving cell proliferation and NE transition [133]	Mevrometostat	Inhibition of EZH2, the core subunit of the histone methyltransferase PRC2 complex	Phase I, NCT03460977 (mevrometostat plus enzalutamide)	In mCRPC patients with a median follow-up of 9.7 months, the PSA_50_ rate was 14.9%, with rPFS of 17.0 months and ORR of 27.3% [170]
	AURKA drives NEPC progression by stabilizing N-MYC protein [171]	Alisertib	Inhibition of AURKA	Phase II, NCT01799278 (alisertib monotherapy)	In mCRPC patients with a median follow-up of 9.7 months, results showed median PFS of 2.2 months and OS of 9.5 months [171]
	BET proteins up-regulate oncogenic genes such as *MYC*, promoting tumorigenesis and progression [172]	ZEN-3694	Inhibition of BET proteins, the epigenetic readers of histone acetylation	Phase Ib/IIa, NCT02711956 (ZEN-3694 plus enzalutamide)	In mCRPC patients, the PSA_50_ rate was 8% and rPFS was 9.0 months, while the AR-low subgroup achieved rPFS of 10.4 months [172]

AR, androgen receptor; mCRPC, metastatic castrate resistant prostate cancer; PI3K, phosphatidylinositol 3-kinase; BAT, bipolar androgen therapy; ARSI, androgen receptor signaling inhibitor; PROTAC, proteolysis-targeting chimeras; HRR, homologous recombination repair; PARP, poly(ADP-ribose) polymerase; LBD, ligand-binding domain; NTD, N-terminal domain; NE, neuroendocrine; EZH2, enhancer of zeste homolog 2; AURKA, Aurora kinase A; H3K27me3, H3K27 trimethylation; BET, bromodomain and extra-terminal domain; GR, glucocorticoid receptor; PSA_50_, 50% decrease in PSA level; PSA_90_, 90% decrease in PSA level; rPFS, radiographic progression-free survival; OS, overall survival; ORR, overall response rate

### AR signaling-related genetic alterations

PCa progression is driven by a spectrum of somatic genetic alterations that interact with AR signaling and influence therapeutic responses. Early events include *TMPRSS2-ERG* fusions, *PTEN* loss, and *SPOP* mutations, while *KMT2C*, *TP53*, *RB1*, and *AR* alterations are more enriched in advanced disease [69,89].

Approximately 50% of PCa exhibit *TMPRSS2-ERG* gene fusion, resulting in *ERG* overexpression that synergizes with *PTEN* deletion (8% to 17% of PCa) to activate the, AKT-pS21-EZH2 pathway, promoting tumor growth and invasion in CRPC [69,90,91]. Zhou et al. [92] reported that *TMPRSS2-ERG* may enhance tumor proliferation via soluble guanylyl cyclase (sGC)–cyclic guanosine monophosphate (cGMP) pathway activation. However, recent studies revealed suppression of sGC activity in CRPC, whereas the sGC agonist riociguat restores cGMP signaling to induce apoptosis, suggesting therapeutic potential in targeting this pathway [93]. *SPOP* mutation could antagonize the *ERG* overexpression pathway by stabilizing ZMYND11, and impair AR ubiquitination-mediated degradation, increasing AR protein stability [41]. *SPOP*-mutant tumors exhibit higher AR signaling dependence than do *ERG*-fusion tumors, correlating with greater endocrine therapy sensitivity and improved clinical outcomes [41,42,94]. *FOXA1* alterations, presented in >34% of metastatic CRPC (mCRPC), promote CRPC by regulating AR bidirectionally: I176M/R261G mutations enhance AR transcriptional activity, whereas D226G mutation impairs AR enhancer recruitment to suppress AR signaling [95], and C-terminal truncations such as P358fs activate the WNT/β-catenin pathway to drive invasion and metastasis [96].

Furthermore, deficiencies of KMT2C, TP53, and RB1 drive tumor lineage plasticity via epigenetic mechanisms. Research has successfully induced adenocarcinoma-to-NEPC (neuroendocrine prostate cancer) conversion in a *PTEN*/*Rb1*/*Trp53* triple-knockout mouse model and adenocarcinoma-to-DNPC (double-negative prostate cancer) transition in *KMT2C*-deficient human patient-derived organoids (PDOs), establishing that genetic mutations promote this transformation through lineage plasticity [89,97]. *KMT2C* loss attenuates ADT-induced *ASPP2* enhancer binding, down-regulating ASPP2 to activate key squamous factor ΔNp63-dependent transdifferentiation, enabling adenocarcinoma-to-AR-negative squamous tumor transition [89]. *TP53*/*RB1* deficiency suppresses AR and luminal gene (like *Krt8*) expression via EZH2-mediated epigenetic silencing [97]. These dual pathways jointly promote lineage plasticity, AR loss, and neuroendocrine (NE) differentiation [98]. AR-negative PCa variants confer intrinsic resistance to endocrine therapies.

### DDR defects and synergy with AR signaling

Genomic defects in DNA damage repair (DDR) pathways occur in >20% mCRPC, primarily affecting homologous recombination repair (HRR)-related genes, such as *BRCA1/2* and *ATM* [99]. The proteins encoded by these genes are involved in repairing DNA double-strand breaks, and HRR deficiency leads to genomic instability and apoptosis [99].

AR can directly promote the expression of DDR-related genes to maintain genomic integrity during cell proliferation [100,101]. Inhibition of AR signaling down-regulates HRR gene expression, increasing DNA damage accumulation [100]. Notably, exposure to ARSIs can induce a “BRCAness” state in HRR-wild-type tumors, conferring HRR-deficient characteristics [102]. Poly(adenosine diphosphate-ribose) polymerases (PARPs) exhibit dual functions that critically bridge DDR and AR pathways. PARPs not only serve as a core component of the DDR pathway but also function as a transcriptional co-regulator [103,104]. For example, PARP-1 enhances the transcriptional activity of the AR through its enzymatic activity-dependent chromatin remodeling capabilities—including modulation of GATA2 recruitment, histone modifications, and chromatin accessibility [103]. For patients with HRR defects, particularly those carrying *BRCA1/2* mutations, dual targeting of AR and DDR (PARP inhibitors) pathways significantly enhances the efficacy of endocrine therapy [105].

### Mutation/activation of AR-related pathways

The AR pathway crosstalk with multiple signaling pathways (including PI3K/AKT, WNT/β-catenin, and NF-κB) can drive treatment resistance [106]. Genetic mutations like *PTEN* deletion/*PIK3CA* mutation [107], and *CTNNB1*/*APC* mutation [106,108], and aberrant up-regulation of inflammatory signals [109], can activate the AR alternative pathways, which disrupts the dynamical balance with AR signaling, driving tumor growth and treatment resistance (Fig. 2).

The PI3K/AKT/mTOR pathway is pivotal in PCa progression. AR can rapidly activate PI3K/AKT nontranscriptionally to provide rapid survival signals and promote tumor glycolysis and lipid metabolism [106,107]. mTOR can directly interact with AR in the nucleus to promote metabolic reprogramming [106,107,110]. However, long-term AR–PI3K signaling exhibits bidirectional crosstalk. On one hand, the PI3K pathway suppresses AR signaling at both protein and translational levels by inhibiting the FOXO1–HER2/3 axis[106,111]. Conversely, AR could reduce AKT activity through the PHLPP/FKBP51/IKKα complex [107]. This equilibrium leads to compensatory activation of the alternate pathway upon single-pathway targeted therapy, resulting in resistance.

AR and NF-κB pathway exhibit positive synergy. NF-κB can promote *AR* and *SRD5A1/2/3* expression through its p50/p65 heterodimer, while NF-κB activates AR transcription via its p52 subunit binding to the N-terminal domain under androgen-deprived conditions, multi-dimensionally activating AR signaling [112–114]. Moreover, AR and AR-V7 can up-regulate MALT1 expression, thereby inducing IκBα phosphorylation and degradation, which subsequently promotes nuclear translocation of the p50/p65 heterodimer and ultimately forms a positive feedback loop to activate the AR–NF-κB pathway [115].

WNT/β-catenin signaling up-regulates AR mRNA expression via recruiting lymphoid enhancer-binding factor 1/T cell factor complex [116]. WNT signaling also enables β-catenin to form transcriptional complexes with AR, enhancing proliferative gene expression (such as *MYC*) [106]. Notably, *CTNNB1* mutation could cause nuclear β-catenin accumulation [117]. Excess β-catenin inhibits AR through 2 distinct mechanisms: direct physical binding and the induction of noncanonical WNT5a/NF-κB signaling, which suppresses AR expression [117]. Conversely, AR reciprocally represses WNT5a expression and noncanonical WNT signaling, establishing a bidirectional inhibitory loop between AR and WNT pathways [117].

The IGF-1–AR positive feedback loop exacerbates AR signaling. TME-derived excessive IGF-1 induces ligand-independent AR activation and nuclear translocation via IGF-1 receptor (IGF-1R), and activation of IGF-1R also increased cytoplasmic and nuclear levels of β-catenin, further enhancing AR-mediated transcription [108,118]. In PCa cells, *IGF1R* is also a direct AR target gene, and AR could elevate IGF-1 activity by suppressing IGF-binding protein 3, creating a self-reinforcing cycle [108,119].

## Spatiotemporal Heterogeneity and Dynamic Evolution of AR Signaling

**Fig. 3.**
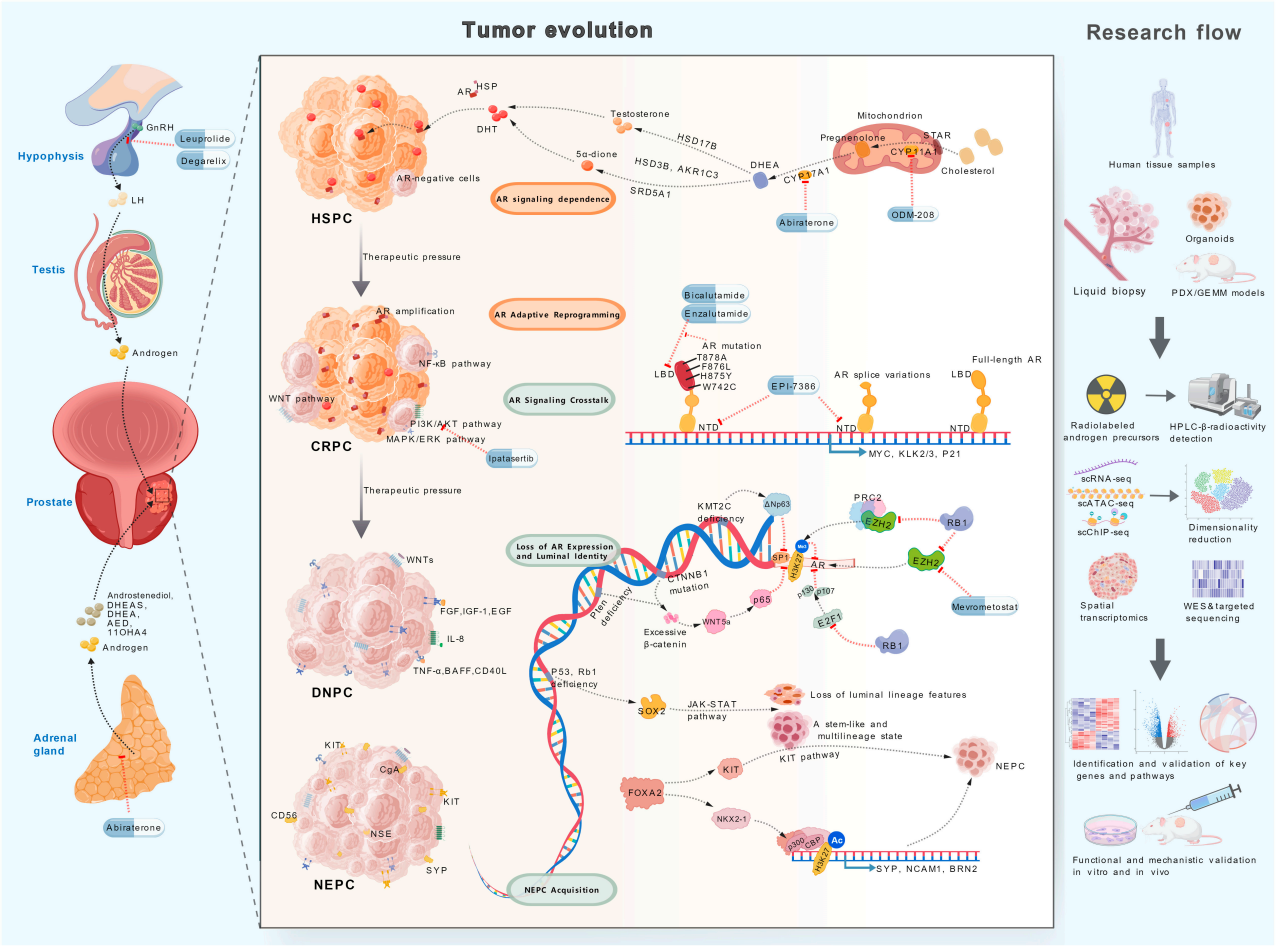
Dynamic progression of PCa to therapy-resistant states and corresponding interventions. Under therapeutic pressure and through accumulating genetic alterations, PCa progresses from hormone-sensitive disease (HSPC) to CRPC, and further to aggressive, AR-independent subtypes like DNPC and NEPC. Key resistance mechanisms—including intratumoral androgen metabolism, AR genetic alterations, and activation of alternative signaling pathways—are highlighted alongside stage-specific therapeutic strategies. To further investigate drug resistance mechanisms, integrated approaches utilizing serial patient samples, liquid biopsies, multi-omics profiling, patient-derived organoids, and preclinical models (like patient-derived tumor xenograft, genetically engineered mouse models) are recommended. The figure was created with BioGDP [163].

The androgen metabolic regulation, AR variants, and multidimensional drug resistance networks described above constitute the core molecular basis of PCa treatment resistance. However, these molecular alterations are neither uniformly distributed throughout the tumor nor static during disease progression. The spatial structural heterogeneity of tumors and their dynamic evolution under therapeutic pressure result in highly complex and individualized resistance mechanisms, posing challenges to clinical management (Fig. 3).

### Spatial heterogeneity

Approximately 80% of PCa exhibit multifocal independent clones, with tumors of identical Gleason scores demonstrating marked differences in histology, molecular characteristics, and prognosis [69,120,121]. This heterogeneity originates from genome instability during the clonal evolution of PCSCs [13]. Large heterogeneity emerges between primary lesions (including intralesional regions) and metastatic sites, which includes regional variations in *AR* expression and function, ultimately leading to heterogeneous treatment responses [8].

Primary lesions are predominantly composed of AR^+^ cells but contain a minority of AR^−^/lo cells (Fig. 3). Liu et al. [122] performed AR immunofluorescence analysis on 11 untreated primary PCa clinical samples and detected an AR^−^ cell subpopulation in all of the samples, with AR^+^ cancer cells representing the majority. The AR^−^/lo cell population lacks AR-mediated differentiation signaling, exhibits enriched stem cell features, demonstrates poor differentiation and high invasiveness, and displays intrinsic resistance to endocrine therapy [8,120]. AR^−^/lo tumor cells drive androgen-independent growth through activation of alternative pathways including PI3K/AKT, NF-κB, and WNT/β-catenin, as well as overexpression of *MYC* and *BCL-2* [120]. AR variants are rare in primary lesions, where *ERG*, *SPOP*, and *PTEN* alterations are more common [69]. AR function also shows tissue-specific heterogeneity: While it primarily promotes proliferation in stromal and luminal epithelial cells, it suppresses invasion and metastasis in basal epithelial cells, contributing to differential endocrine therapy responses [123].

Metastatic lesions often exhibit monoclonal origins, with higher molecular similarity between lesions than between regions within primary tumors [69]. However, metastatic lesions demonstrate higher frequencies of both AR pathway hyperactivation and AR^−^/lo phenotypes compared to primary lesions [124–126]. Labrecque et al. [127] reported that AR^+^ metastases are most prevalent, while about 14.7% of patients with multiple metastases exhibit interlesional molecular phenotypic disparities. *AR* amplifications/mutations frequently occur synchronously across multiple metastatic sites, whereas AR^−^/lo phenotypes display pronounced heterogeneity [126]. Concurrently, metastatic lesions also show reduced expression of androgen synthases like CYP11A1 and CYP17A1 [25]. Metastatic lesions (particularly mCRPC) are enriched for *PTEN*, *TP53*, and *RB1* deletions compared to primary tumors, forming the basis of highly aggressive subtypes [97,126,128]. During endocrine therapy resistance, metastatic lesions develop AR alterations and undergo phenotypic transitions from AR^+^NE^−^ cells to AR^−^ (DNPC) or NE phenotypes (NEPC) [120]. In deceased mPCa patients, studies suggest that 65% harbor coexisting AR^+^/NE^−^ and AR^+^/NE^+^ cells across different metastases [129].

### Temporal heterogeneity

Temporal heterogeneity refers to the clonal evolution of molecular features in tumor cells during disease progression and under therapeutic pressure. At the early stage of PCa, AR alternations are not common [69]. Certain mutations, like *SPOP*, can lead to aberrant AR signaling activation [94]. A small part of PCSCs originating from mutations in normal basal/luminal cells exhibit an AR^−^/lo phenotype [13], and studies suggest that primary lesions harbor adaptive CRPC-like cell subpopulations early on, which persistently expand during progression [7,130]. Taavitsainen et al. [131] utilized single-cell RNA sequencing and single-cell assay for transposase-accessible chromatin with sequencing to identify preexisting and persistently surviving drug-resistant cell subpopulations in pretreatment enzalutamide models. These subpopulations exhibited open chromatin sites enriched with transcription factor motifs including MYC and E2F1, and their accessibility patterns remained unperturbed by drug treatment, indicating that intrinsic epigenetic features may mediate resistance [131].

Endocrine therapy reshapes the AR pathway and drives resistance through dual mechanisms of induction and selection. On one hand, ADT and ARSIs are more effective against *SPOP*-mutated and AR^+^ subpopulations [41,69], allowing AR^−^/lo cells to gradually dominate in CRPC and accelerate resistance development [132]. Additionally, AR elimination abrogates the tumor-suppressive role of AR in certain cell types (like epithelial basal intermediate cells), enhancing tumor aggressiveness [123]. On the other hand, the therapeutic pressure prompts AR^+^ tumor subpopulation adaptation via enhanced androgen synthesis, activation of noncanonical AR or alternative pathways, or down-regulation of *AR* expression through intricate genetic and epigenetic mechanisms [9,35,67,88]. In *CTNNB1* mutation tumors, the excessive accumulation of β-catenin could repress AR expression through WNT5a/NF-κB signaling [117]. PTEN deficiency could synergistically enhance the oncogenic activity of β-catenin and collectively drive AR-independent CRPC [117]. In RB1-deficient tumors, the loss of RB1 function leads to aberrant activation of both E2F and EZH2 [97]. EZH2 mediates PRC2-dependent H3K27me3 deposition at the AR promoter, and E2F1 directly binds the AR promoter alongside pocket proteins p107 and p130, together repressing AR expression [124,133]. Additionally, the deficiency of epigenetic checkpoint KMT2C could up-regulate ΔNp63, which in turn represses SP1, a crucial transcriptional regulator of the AR gene [89]. Under sustained ADT and ARSI pressure, AR^−^/lo cells become enriched in CRPC (with AR detectable in only about 40% of mCRPC cells in some studies) [124]. When AR expression is lost in the absence of NE marker expression, PCa progresses to the DNPC. There is a theory proposing that DNPC is an intermediate stage between AR-positive adenocarcinoma and NEPC, while some studies regard both DNPC and NEPC as distinct terminal subtypes of CRPC [134].

Concurrently, accumulation of DDR-related mutations along with increased clonal loss of tumor suppressor genes such as *TP53*/*RB1* drives genome instability and enhances lineage plasticity [67,135–137]. In the early stage of NEPC, following TP53/RB1 loss, SOX2-driven ectopic JAK–STAT activation promotes the loss of luminal identity and the acquisition of a stem-like, multilineage state in tumor cells [138]. Under ADT pressure, FOXA2 and ASCL1 are up-regulated. ASCL1 promotes NE lineage transition from KRT8^+^ luminal epithelial cells, while FOXA2 directly promotes the KIT signaling pathway within tumor cells to sustain NEPC proliferation and tumor growth [139,140]. In addition, a recent study found that FOXA2 could induce NKX2-1 expression, and they cooperatively recruit p300/CBP to NE enhancers to catalyze H3K27ac, thereby activating the expression of NE marker genes such as *SYP*, *NCAM1*, and *BRN2*, driving NEPC transdifferentiation [98].

## Challenges and Future Directions

The development of therapeutic resistance in PCa is driven by the complex interplay of intratumoral androgen anabolic reprogramming, AR alterations, multidimensional crosstalk networks, and genetic lineage reprogramming, all of which exhibit pronounced spatiotemporal heterogeneity. A primary challenge lies in moving beyond static research paradigms. Current research predominantly relies on static samples (including radical prostatectomy specimens or metastatic biopsies at limited time points), which fails to capture the real-time coevolution of the intratumoral androgen microenvironment and genomic alterations under therapeutic pressure [69,120]. Overcoming these barriers requires a multidimensional understanding of AR signaling adaptation, stem cell plasticity, and NE transformation [141]. Future studies should emphasize the use of serial patient samples, liquid biopsies, genetically engineered mouse models, and PDOs, integrated with high-resolution single-cell and spatial multi-omics technologies [142].

Current research extensively focuses on the transcriptional/epigenetic regulatory functions of AR signaling and targeting alternative pathways (like PI3K-AKT). Future investigations should actively explore the therapeutic potential of cotargeting the AR signaling with other alternative pathways, such as NF-κB, and focus on interactive nodes beyond AR itself [143]. The impact of epigenetics on androgen metabolism is largely underexplored. Future work should investigate whether and how androgens serve as substrates to influence epigenetics, thereby silencing tumor suppressor genes [8]. Deeper exploration into how posttranslational modifications reshape the expression profile of androgen metabolic enzymes is needed to elucidate the cross-talk between metabolism and epigenetics [38]. The metabolic regulation of the immune microenvironment is another crucial direction. PCa is considered an “immunologically cold” tumor, and immunotherapy has yet to achieve breakthrough success in this area [144]. Evidence suggests that endocrine therapy and the intratumoral immune microenvironment can influence each other [4,145]. Single-cell sequencing is a powerful tool for dissecting interactions between tumor cells and immune/stromal cells, and for identifying tumor subtypes most likely to benefit from immunotherapy [145]. Future research needs to elucidate whether intratumoral androgen metabolism/pathways can regulate the function of specific immune cells (like regulatory T cells and myeloid-derived suppressor cells) and whether immune cell-derived cytokines (like IL-10) can feedback to affect tumor cell androgen synthesis/signaling, forming a “metabolism–immune” axis of resistance. Furthermore, there exist potential interactions between immune responses, circadian rhythms, and cellular senescence, and future studies should also explore their potential cross-talk with androgen metabolic pathways in PCa [146]. Proteomic studies often overlook proteins encoded/regulated by traditionally defined “noncoding” genomic regions, which may play important roles in tumor biology [147]. Future research should explore the interactions and links between these noncanonical proteins and the androgen pathway in PCa.

To advance from common treatment toward precision intervention, there is an urgent need to develop novel biomarkers and combination strategies. The predictive value of existing biomarkers like PSA is limited, necessitating more comprehensive subtyping tools and dynamic monitoring systems. Future research should leverage longitudinal liquid biopsy techniques, such as the combined analysis of circulating tumor DNA epigenetic profiles and androgen metabolite profiles, to noninvasively monitor the dynamic reprogramming of intratumoral androgen synthesis pathways and the evolutionary trajectory of emerging resistant clones [148]. Direct dynamic monitoring of androgen metabolism via tritium-labeled androgen precursors and high-performance liquid chromatography (HPLC)–β-radioactivity detection can further uncover genetic regulators of intratumoral androgen dynamics [149]. Simultaneously, the application of spatial multi-omics technologies enables in situ analysis of the TME, helping to reveal the spatial colocalization of androgen metabolism “hotspots” with cells harboring specific genetic variants, thereby identifying “cellular neighborhood effects” that drive resistance [58,150]. In therapeutic testing, bioprinting technology and PDOs can serve as functional biomarker platforms to directly test tumor sensitivity to novel targeted drugs in vitro, combined with their genetic variant profiles, enabling the simultaneous development of “drug–biomarker” pairs [151]. Furthermore, artificial intelligence models integrating patient multi-omics data can predict compensatory resistance pathways most likely to be activated, providing a rationale for future therapeutic testing of relevant combination strategies [152].

The seeds of therapeutic resistance may be sown early, even at diagnosis. Future research needs to focus on earlier stage. Challenges such as the lack of standardized biomarkers for the early detection of lineage plasticity transitions hinder timely clinical intervention. Multi-omics analysis (like digital pathology combined with single-cell sequencing) of tumor biopsies could be used to develop a “therapy resistance risk score” to identify high-risk patients, prompting consideration of adjuvant targeted interventions following radical treatment to prevent the expansion of resistant clones in micrometastases [153]. Additionally, novel AR regulatory mechanisms should be explored, focusing on the activation mechanisms of noncanonical androgen signaling (like membrane-associated AR) in low-androgen environments, laying the groundwork for developing novel AR functional inhibitors [154]. For advanced tumors, particularly aggressive subtypes like NEPC driven by epigenetic dysregulation and metabolic reprogramming, active investigation should focus on lineage-specific targets and potential combination strategies [89,139]. Butler et al. [155] discovered that the glycosylated protein glypican-3 is specifically highly expressed on the surface of NE cells, while it is scarcely expressed in hormone-sensitive prostate cancer. Knockdown of glypican-3 inhibits the proliferation and survival of NE cells, making it a potential target for the precise diagnosis and treatment of NEPC [155]. The accumulation of DNA replication mutations and genomic instability under therapeutic pressure in advanced disease may render cellular senescence a potential therapeutic target [156]. PARP inhibitors can exacerbate replication stress in HRR-deficient cancer cells and may potentially induce senescence in PCa [157]. Aging is associated with decreased melatonin secretion and circadian rhythm disruption, which collectively exacerbate oxidative stress and inflammation, promoting tumor progression [158]. Advanced PCa exhibits marked glycolipid metabolic abnormalities. DNPC exhibits aberrant activation of SREBP1c-driven fatty acid metabolic pathways and promotes growth via the MAPK/ERK (extracellular signal-regulated kinase) pathway [89], whereas NEPC demonstrates enhanced glycolysis to sustain AR-independent survival [159]. Tumor metabolic reprogramming may offer therapeutic targets for advanced aggressive PCa [160]. Additionally, lysosomal function is closely linked to cellular metabolism [161]. Future research needs to explore the link between lysosomal function and glycolipid metabolism regulation in late-stage disease and its potential as a therapeutic target.

Integrating diverse data streams—from genomic, transcriptomic, epigenomic, and metabolic analyses—could improve the selection and adaptation of personalized combination therapies throughout the disease course, moving beyond static biopsies to a continuous management model [162].

## Conclusion

This review systematically synthesizes the multidimensional drug resistance network in PCa, encompassing intratumoral androgen metabolism, AR genetic alterations, pathway crosstalk, and spatiotemporal heterogeneity. It underscores the necessity of cotargeting androgen metabolic reprogramming and AR-interacting pathways via precision interventions, coupled with dynamic monitoring technologies to track and counteract the evolution of resistant clones. Future efforts should focus on identifying key targets within the androgen signaling axis and developing synergistic therapeutic and translational strategies to suppress lineage plasticity, thereby overcoming resistance barriers in PCa.
