# Supplementary material for: Intratumoral Androgens and Genetic Variants Driving Therapy Resistance in Prostate Cancer
Source: Research (Wash D C). 2026 Feb 24;2026:1128. doi: 10.34133/research.1128 (PMC12929811; doi:10.34133/research.1128)
Supplement: Supplementary 1 — Table S1 [file research.1128.f1.docx]

| Supplementary table 1\| The characteristics of core included studies. | | | | | | |
| --- | --- | --- | --- | --- | --- | --- |
| Author | Year | Native English | Review/article | Basic/clinical | Journal | PMID/DOI |
| Rhee et al | 2024 | Yes | Review | Basic | Oncogene | 39369165 |
| Jamroze et al | 2021 | Yes | Review | Basic | Cancer Letters | 34118355 |
| Watson et al | 2015 | Yes | Review | Basic | Nature Reviews Cancer | 26563462 |
| Page et al | 2006 | Yes | Article | Basic | The Journal of Clinical Endocrinology and Metabolism | 16882745 |
| Zhou et al | 2021 | No | Review | Basic | Oncogene | 33750894 |
| Kim et al | 2024 | No | Article | Basic | Nature Communications | 38336745 |
| Geller et al | 1978 | No | Article | Clinical | Cancer Biology & Medicine | 87401 |
| Lubik et al | 2013 | Yes | Article | Basic | Endocrine-Related Cancer | 23319492 |
| Shiota et al | 2023 | No | Review | Basic | Urologic Oncology | 36376200 |
| Shafiee et al | 2021 | Yes | Article | Basic | Epigenetics | 32660355 |
| Liu et al | 2023 | No | Review | Basic | Frontiers in Oncology | 36741721 |
| Snaterse et al | 2024 | Yes | Article | Basic | The Journal of Steroid Biochemistry and Molecular Biology | 38104728 |
| Barnard et al | 2020 | No | Review | Basic | The Journal of Steroid Biochemistry and Molecular Biology | 31672619 |
| Zhang et al | 2022 | No | Review | Basic | International Journal of Molecular Sciences | 36362304 |
| He et al | 2007 | Yes | Article | Basic | The Prostate | 17626250 |
| Arakawa et al | 2012 | No | Article | Basic | Biochemical Pharmacology | 22864060 |
| Brennen et al | 2021 | Yes | Article | Basic | JCI Insight | 33724955 |
| Cai et al | 2011 | Yes | Review | Basic | Endocrine-Related Cancer | 21712345 |
| Hofland et al | 2010 | Yes | Article | Basic | Cancer Research | 20086173 |
| Du et al | 2017 | Yes | Article | Basic | The Journal of Steroid Biochemistry and Molecular Biology | 27345701 |
| Barnard et al | 2018 | No | Article | Basic | Journal of Steroid Biochemistry and Molecular Biology | 29936123 |
| Hou et al | 2022 | No | Article | Basic | Cell Reports Medicine | 35492874 |
| Kong et al | 2018 | Yes | Article | Basic | The Journal of Biological Chemistry | 30089652 |
| Škara et al | 2021 | No | Review | Basic | Cancers | 34572923 |
| Xue et al | 2020 | No | Review | Basic | Frontiers in Oncology | 32974183 |
| Nguyen et al | 2023 | Yes | Article | Basic | Nature Communications | 37296155 |
| Chen et al | 2025 | No | Article | Basic | Nature Cancer | 40360905 |
| Bernasocchi et al | 2021 | Yes | Article | Basic | Nature Communications | 33531470 |
| Zhe et al | 2014 | No | Article | Basic | Cancer research | 25252916 |
| Pinski et al | 2011 | Yes | Article | Basic | The Prostate | 21456071 |
| Nakazawa et al | 2022 | Yes | Article | Clinical | The Prostate | 34783071 |
| Cai et al | 2011 | Yes | Article | Basic | Cancer Cell | 22014572 |
| Zhou et al | 2021 | No | Article | Basic | Clinical and Translational Medicine | 34185414 |
| Lubik et al | 2011 | Yes | Article | Basic | Cancer Research | 21747118 |
| Vasileiou et al | 2020 | Yes | Article | Basic | Journal of B.U.ON.: official journal of the Balkan Union of Oncology | 32521918 |
| Ahtziri et al | 2024 | No | Article | Basic | Current Molecular Pharmacology | 39806979 |
| Di et al | 2021 | Yes | Article | Basic | Cell Death & Disease | 33500395 |
| Cui et al | 2023 | Yes | Article | Basic | The Journal of Clinical Investigation | 37009898 |
| Le et al | 2022 | Yes | Article | Basic | American Journal of Cancer Research | 35141012 |
| Qin et al | 2024 | Yes | Article | Basic | Cell Reports | 38181788 |
| Patel et al | 2020 | Yes | Article | Basic | Cancer Research | 31719098 |
| Shorning et al | 2020 | Yes | Review | Basic | International Journal of Molecular Sciences | 32630372 |
| Tyagi et al | 2024 | Yes | Review | Basic | Pharmacology & Therapeutics | 39491756 |
| Markowski et al | 2021 | Yes | Article | Clinical | European Urology | 32624280 |
| Isaacsson et al | 2025 | Yes | Meeting Abstract | Clinical | Journal of Clinical Oncology | DOI:10.1200/JCO.2025.43.5_suppl.178 |
| Weiss et al | 2024 | Yes | Article | Basic | International Journal of Cancer | 38602058 |
| Cotter et al | 2022 | Yes | Review | Basic | The Prostate | 35657155 |
| Waltering et al | 2009 | No | Article | Basic | Cancer Research | 19808968 |
| Borgmann et al | 2018 | Yes | Article | Clinical | European Urology | 28851578 |
| Li et al | 2025 | No | Review | Basic | Frontiers in Oncology | 40008000 |
| Bohl et al | 2005 | Yes | Article | Basic | Proceedings of the National Academy of Sciences of the United States of America | 15833816 |
| Korpal et al | 2013 | Yes | Article | Basic | Cancer Discovery | 23842682 |
| Cato et al | 2019 | Yes | Article | Basic | Cancer Cell | 30773341 |
| Danielli et al | 2025 | Yes | Review | Basic | Expert Review of Anticancer Therapy | 40089934 |
| He et al | 2021 | Yes | Article | Clinical | Nature Medicine | 33664492 |
| Fan et al | 2018 | No | Article | Basic | Proceedings of the National Academy of Sciences of the United States of America | 29712835 |
| Koh et al | 2002 | Yes | Article | Basic | The Prostate | 12242730 |
| Teramoto et al | 2024 | Yes | Article | Basic | International Journal of Molecular Sciences | 39000396 |
| Marek et al | 2024 | Yes | Article | Basic | Vaccines | 39591176 |
| Zammit et al | 2025 | Yes | Article | Basic | Journal of the American Chemical Society | 40490871 |
| Xue et al | 2024 | Yes | Article | Basic | Molecular Pharmaceutics | 39388218 |
| Ha et al | 2025 | No | Article | Basic | European Journal of Medicinal Chemistry | 39577229 |
| Lin et al | 2021 | No | Review | Basic | Cancers | 34298665 |
| Fizazi et al | 2024 | Yes | Article | Clinical | NEJM evidence | 38320513 |
| Rathkopf et al | 2025 | Yes | Article | Clinical | Annals of Oncology: Official Journal of the European Society for Medical Oncology | 39293515 |
| Petrylak et al | 2024 | Yes | Meeting Abstract | Clinical | Journal of Clinical Oncology | DOI:10.1200/JCO.2024.42.16_suppl.5011 |
| Kyriakopoulos et al | 2024 | Yes | Meeting Abstract | Clinical | Annals of Oncology | DOI:10.1016/j.annonc.2024.08.1722 |
| Raith et al | 2023 | No | Review | Basic | International Journal of Molecular Sciences | 36768610 |
| De et al | 2025 | Yes | Article | Clinical | European Urology | 39884884 |
| Piombino et al | 2024 | No | Review | Basic | International Journal of Molecular Sciences | 38731844 |
| Fallah et al | 2024 | Yes | Article | Clinical | Journal of Clinical Oncology | 38127780 |
| Park et al | 2021 | Yes | Review | Basic | Oncogene | 34349243 |
| Schweizer et al | 2024 | Yes | Meeting Abstract | Clinical | Journal of Clinical Oncology | DOI:10.1200/JCO.2024.42.16_suppl.5061 |
| Beltran et al | 2019 | Yes | Article | Clinical | Clinical Cancer Research | 30232224 |
| Aggarwal et al | 2020 | Yes | Article | Clinical | Clinical Cancer Research | 32694156 |
| Zoma et al | 2021 | No | Article | Basic | Nature Communications | 34230470 |
| Aurilio et al | 2020 | Yes | Review | Basic | Cells | 33321757 |
| Zhang et al | 2025 | Yes | Article | Basic | Cancer Research | 39388307 |
| Guo et al | 2025 | No | Article | Basic | Cancer cell | 40280125 |
| Lu et al | 2025 | No | Article | Basic | Nature Genetics | 40691407 |
| Gui et al | 2019 | No | Article | Basic | Proceedings of the National Academy of Sciences of the United States of America | 31266892 |
| McKay et al | 2024 | Yes | Review | Basic | Cancer Treatment Reviews | 38613872 |
| Adzavon et al | 2025 | Yes | Review | Basic | Nature Reviews Urology | 39375467 |
| Al-Rashidi et al | 2023 | Yes | Review | Basic | Pharmacological Research | 37075872 |
| Chang et al | 2023 | Yes | Article | Basic | International Journal of Molecular Sciences | 37047218 |
| Deng et al | 2022 | No | Article | Basic | Nature Cancer | 36065066 |
| Han et al | 2022 | No | Article | Basic | Cancer Cell | 36332622 |
| Tang et al | 2022 | Yes | Review | Basic | Seminars in Cancer Biology | 34844845 |
| Li et al | 2018 | Yes | Article | Clinical | Nature Communications | 30190514 |
| Cyrta et al | 2022 | Yes | Article | Clinical | The Journal of Pathology | 35220606 |
| Romero et al | 2024 | No | Article | Basic | Nature Cancer | 39394434 |
| Ku et al | 2017 | Yes | Article | Basic | Science (New York, N.Y.) | 28059767 |
| Roudier et al | 2025 | Yes | Article | Clinical | The Journal of Clinical Investigation | 40493417 |
| Cheng et al | 2022 | Yes | Article | Clinical | European Urology | 35058087 |
| Taavitsainen et al | 2021 | No | Article | Basic | Nature Communications | 34489465 |
| Patel et al | 2024 | Yes | Article | Basic | European Urology Focus | 39613543 |
| Jayalath et al | 2023 | Yes | Review | Clinical | JAMA network open | 36449294 |
| Butler et al | 2023 | Yes | Article | Basic | The Journal of Pathology | 36752189 |
| Zhang et al | 2023 | No | Article | Basic | The Journal of Clinical Investigation | 38099500 |
| Matos et al | 2025 | No | Review | Basic | Cancer letters | 41319862 |
| Horestani et al | 2025 | No | Article | Basic | Cancers (Basel) | 40075640 |
